# Nitazenes—heralding a second wave for the UK drug-related death crisis?

**DOI:** 10.1016/S2468-2667(24)00001-X

**Published:** 2024-01-12

**Authors:** Adam Holland, Caroline S Copeland, Gillian W Shorter, Dean J Connolly, Alice Wiseman, John Mooney, Kevin Fenton, Magdalena Harris

**Affiliations:** Faculty of Public Health Drugs Special Interest Group, London, UK; School of Psychological Science; School of Population Health Sciences; University of Bristol, Bristol BS8 2BN, UK; Department of Public Health, Faculty of Public Health and Policy, London School of Hygiene and Tropical Medicine, London, UK; Institute of Pharmaceutical Science, King’s College London, London, UK; National Programme on Substance Abuse Deaths, King’s College London, London, UK; Faculty of Public Health Drugs Special Interest Group; Drug and Alcohol Research Network, School of Psychology, Queen’s University Belfast, Belfast, UK; Faculty of Public Health Drugs Special Interest Group; University of Bristol, Bristol BS8 2BN, UK; Department of Public Health, Faculty of Public Health and Policy, London School of Hygiene and Tropical Medicine, London, UK; National Addiction Centre, Institute of Psychiatry, Psychology and Neuroscience, King’s College London, London, UK; Faculty of Public Health Drugs Special Interest Group; Association of Directors of Public Health, London, UK; Faculty of Public Health Drugs Special Interest Group; Public Health Directorate, NHS Grampian, Aberdeen, UK; School of Medicine, Medical Sciences and Nutrition, University of Aberdeen, Aberdeen, UK; Faculty of Public Health, London, UK; Faculty of Public Health Drugs Special Interest Group; University of Bristol, Bristol BS8 2BN, UK; Department of Public Health, Faculty of Public Health and Policy, London School of Hygiene and Tropical Medicine, London, UK

The UK is in the midst of a drug-related crisis.^1^ Likely contributory factors include: disinvestment in drug treatment, harm reduction, and public services; changing patterns of socioeconomic deprivation; increasing poly-drug use; and an ageing cohort of people who use heroin.^1^ The UK crisis has not reached the scale of that in the US where 106 699 overdose deaths were reported in 2021.^2^ The latest wave of the crisis in the US has been driven primarily by fentanyl and other synthetic opioids, which were implicated in 88% of these deaths.^2^

Highly potent synthetic opioids have not played such a prominent role in rising deaths among people who use drugs in the UK. Global drug markets are, however, rapidly evolving. Of particular concern is the emergence of nitazenes: a class of synthetic opioids developed by the pharmaceutical industry in the 1950s but never approved as medicines.^3^ Like other opioids, nitazenes can cause fatal respiratory depression, and some are hundreds of times more potent than heroin (table).^3^ In the UK, nitazenes have been detected in substances sold as other opioids, benzodiazepines, and cannabis products.^4^ This means many consumers are using nitazenes inadvertently, unaware of the risks they face.

The UK National Crime Agency reported 54 deaths in the last six months where nitazenes were detected in post-mortem toxicology.^5^ This is likely the tip of the iceberg, as many tests are still in process and emerging drugs are not routinely tested for. Other European countries, particularly the Baltic states, have also reported increasing numbers of deaths related to nitazenes.^6^

Potent synthetic drugs offer clear benefits for producers. They can be manufactured rapidly and inexpensively, without relying on illicit crops susceptible to eradication. Most of the heroin in Europe originates in Afghanistan. The Taliban recently prohibited opium cultivation, leading to a dramatic reduction in production.^7^ The resultant gap in the market could lead to nitazenes becoming increasingly prominent across Europe. Enforcement and stricter scheduling cannot be expected to solve a problem that has developed in the context of, and might have been exacerbated by, interdiction and prohibition. Without concerted action, nitazenes could devastate communities of people who use a range of drugs, including those who use drugs infrequently or source benzodiazepines and opioid painkillers from the internet.

Drugs services must adapt to improve service accessibility and acceptability, with same day prescribing of medications for opioid dependence, including injectable options, and medications for benzodiazepine dependence. Existing naloxone programmes should be expanded, and additional services should provide both intramuscular and intranasal formulations to people who use drugs or that might encounter overdoses.

Supplementary funding should be made available to introduce additional harm reduction services, currently unavailable in the UK, which would give people who use drugs further opportunities to keep themselves safe. First, drug checking services, where the public can test substances to ensure they do not contain adulterants. These services are evidenced to reduce riskier drug-taking practices and provide invaluable intelligence about the drug market.^8^ Second, overdose prevention centres, where people can use their own drugs in a safe environment with providers present to intervene in any overdoses. Despite their benefits, widespread calls for their implementation are still resisted by the UK Government.^9^ Although dedicated sites should be introduced in areas experiencing high levels of drug use, tolerated use spaces in established drugs services and hostels would optimise accessibility. Third, models of safer supply, which range from the medical provision of prescribed opioids to activists testing and distributing drugs. Safer supply initiatives arose in response to the toxic drug supply in North America and are recognised as a crucial tool to reduce overdose risks.^10^ These interventions could be most beneficial for people not in contact with specialist drug services. It is therefore crucial that services are low-threshold and accommodate the needs of people who use a variety of drugs, given indications of widespread contamination.

Unfortunately, high levels of structural and social stigma pose a key barrier to drug policy innovation. Decision makers often oppose initiatives which the media and public might view as condoning drug use. Similar arguments were used to oppose the introduction of other harm reduction interventions such as needle and syringe programmes that subsequently saved countless lives.^9^ Governments today must again find the political capital to facilitate the policies needed to prevent many more deaths. Actions taken now will not have been taken soon enough.

AH is co-chair and GWS, DJC, AW, JM, and MH are members of the Faculty of Public Health Drugs Special Interest Group. AH is conducting a PhD funded by the Medical Research Council (grants MR/W006308/1 and MR/X018636/1); he volunteers for the Loop (a drug checking organisation); is a member of the Drug Science Enhanced Harm Reduction Working Group (EHRWG); and in the last three years was Associate Director of International Doctors for Healthier Drug Policies. CSC is a co-opted member of the Advisory Council on the Misuse of Drugs Novel Psychoactive Substances Sub-Committee. GWS is a member of the Drug Science EHRWG and is funded by the National Institute for Health and Care Research to develop evaluation and service models for overdose prevention centres in the UK. MH is a member of the Drug Science EHRWG and has received payment from Ethypharm for a conference presentation.

## Figures and Tables

**Table:** The relative potency of fentanyl and selected nitazenes in comparison to heroin^2,3^

	Relative potency to heroin
Heroin	1
Fentanyl	50
Metonitazene	50
Protonitazene	100
Isotonitazene	250
Etonitazene	500
